# Unveiling the Micro-Mechanism of Functional Group Regulation for Enhanced Dielectric Properties in Novel Natural Ester Insulating Oil TME-C_10_

**DOI:** 10.3390/molecules30071431

**Published:** 2025-03-24

**Authors:** Min Chen, Tao Zhang, Jinyuan Zhang, Chunyi Liu, Dong Chen, Jin Zhang

**Affiliations:** 1 College of Electrical Engineering and New Energy, China Three Gorges University, Yichang 443002, China; 2 College of Physics and Electronic Information Engineering, Hubei Engineering University, Xiaogan 432000, China; 3 Hubei Provincial Key Laboratory for Operation and Control of Cascaded Hydropower Station, China Three Gorges University, Yichang 443002, China; 4 State Grid Hubei Electric Power Co Yichang Power Supply Company, Yichang 443000, China

**Keywords:** natural ester insulating oil, density functional theory, electronic properties, dielectric strength

## Abstract

The functional groups in the molecular structure of natural ester insulating oil have a significant impact on its physicochemical and electrical properties. This article takes the novel synthetic ester TME-C_10_ and traditional natural ester GT molecules as research objects, and based on density functional theory (DFT) calculations, systematically explores the micro-mechanism of the effects of C=C double bonds, ester groups (-COOC), and β-H groups on the performance of insulating oils. The results show that the chemical stability and anti-aging ability of the TME-C_10_ molecule are significantly improved by eliminating the C=C double bond and β-H group. The electronic behavior of the TME-C_10_ molecule is mainly controlled by the ester group (-COOC), while the GT molecule is significantly affected by the unsaturated C=C double bond, resulting in a significant difference in the mode of electronic transition between the two molecules: the TME-C_10_ molecule shows the $n\to \sigma^{*}$ transition, while the GT molecule is the $\pi \to \pi^{*}$ transition. In addition, the HOMO orbital energy level, electron transition energy, and ionization energy of the GT molecules are lower than those of the TME-C_10_ molecules. Under the action of an external electric field, the TME-C_10_ molecules exhibit excellent dielectric properties. In summary, the TME-C_10_ molecules not only overcome the aging defects of traditional natural ester insulating oils, but also possess excellent insulation properties, making it a new type of insulating oil material with broad application prospects.

## 1. Introduction

As a new type of high natural point, environmentally friendly liquid dielectric, natural ester insulating oil has been widely and maturely used in the field of distribution transformers, and gradually extended to 220 kV and above large power transformers [1]. Although natural ester insulating oils have many advantages, they cannot completely replace mineral insulating oils at present. Due to the differences in molecular structure, compared with mineral insulating oil, natural ester insulating oil has the disadvantages of weak oxidation resistance [2], high kinematic viscosity [3,4] and a high condensation point [5,6], so natural ester insulating oil is still seldom used in high-voltage power transformers in the power system. The raw material used in natural ester insulating oil is mainly based on vegetable oils, such as soybean oil, rapeseed oil, olive oil, camellia oil, sunflower oil, corn oil, linseed oil, and castor oil, etc., and their main components are triglyceride molecules. The types and contents of fatty acids in different vegetable oils are different, and the differences are mainly reflected in the origin of the carbon atoms in the three ester groups of the natural esters, as well as the degree of unsaturation and the degree of conjugation of unsaturated bonds. During the use and research of natural ester insulating oils, it has been found that the unsaturated C=C double bonds, ester groups, β-H in glycerol molecules, and the length of fatty acid carbon molecule chains in the molecular structure of ester insulating oils are the main factors affecting the physicochemical and electrical properties of natural ester insulating oils [7]. For example, the high content of long-chain saturated fatty acids leads to the high viscosity of natural ester insulating oils [8]; the unsaturated C=C double bond and β-H in the triglyceride molecule lead to the deterioration of the aging resistance of the natural ester insulating oils [9,10,11]; and the influence of ester groups in the molecular structure of the triglyceride molecule makes the lightning breakdown voltage of natural ester insulating oils significantly lower than that of mineral oils [12].

To improve the comprehensive performance of natural ester insulating oils, scholars have completed much research [13,14]. The published research methods in these published reports have been summarized [15], which mainly include several aspects: the use of additives, such as adding antioxidants to natural ester insulating oils [16]; adding nanomaterials into natural ester insulating oils in a certain proportion for modification [17,18]; chemically modifying the natural ester insulating oils [19]; and altering the design of power transformers, such as optimizing the cooling system. Although these methods have been proved to be effective in improving the performance of natural ester insulating oils, they do not take into account this aspect of the modification method, which is to reduce or eliminate as much as possible the effect of unsaturated C=C double bonds, ester groups, and β-H in the glycerol molecule on the comprehensive performance of natural ester insulating oils through the modification of the molecular structure of the insulating oils. For this reason, scholars began to consider improving the performance defects of natural ester insulating oils from the molecular structure perspective, such as using molecular modification (partial hydrogenation) [20] or using epoxidation techniques [21,22] to reduce the unsaturated C=C double bonds in vegetable oil-based insulating oils. In addition, in order to eliminate the β-H bonds in glycerol molecules, Bertrand and Lauzevis [23] used 2-ethyl-1-hexanol instead of glycerol.

The oil base of traditional natural ester insulating oil is soybean oil, and its fatty acids include stearic acid, oleic acid, linoleic acid, and linolenic acid, etc. Natural ester insulating oil is a compound formed through the esterification reaction of one glycerol molecule with three fatty acid molecules, such as the glyceryl trioleate molecule (GT). To eliminate functional groups such as C=C double bonds and β-H in traditional natural ester insulating oils, this study employs low-melting-point and cost-effective trimethylolpropane (TMP) to replace glycerol, and decanoic acid (C_10_H_20_O_2_) extracted from coconut oil to substitute for long-chain fatty acids. These compounds are used in an esterification dehydration reaction to prepare a new type of natural ester insulating oil, designated as TME-C_10_. Using quantum chemistry theory, the reaction pathway of the esterification process between TMP and C_10_H_20_O_2_ is calculated to evaluate the feasibility of the new synthetic ester reaction. Furthermore, a comparative analysis is conducted to examine the micro-mechanisms by which typical functional groups (such as C=C double bonds, ester groups, and β-H) in the structures of the new synthetic ester molecules and traditional triglyceride molecules influence the properties of natural ester insulating oils. Additionally, the dielectric strength of the new synthetic ester insulating oils is assessed.

## 2. Results

### 2.1. Analysis of the IRC for the Synthesis of New Natural Esters

**Figure 1:**
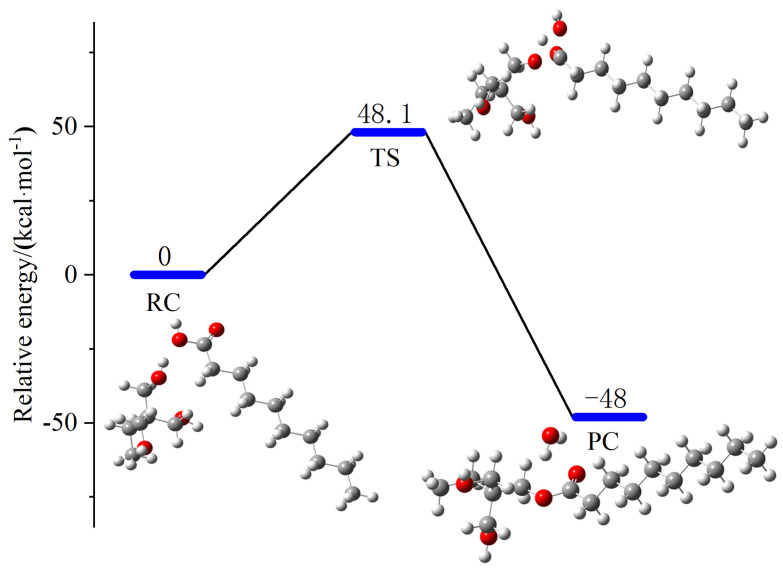
Reaction paths and reaction energy barriers for esterification and dehydration of medium-chain fatty acid ester molecules.

**Figure 2:**
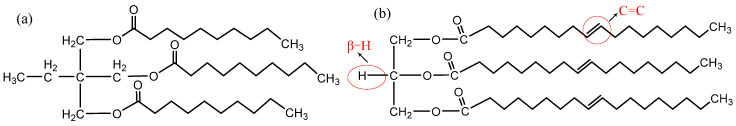
Molecular structure formula: (**a**) TME-C_10_; (**b**) GT.

C_10_H_20_O_2_ and TMP were esterified and dehydrated to synthesize medium-chain fatty acid ester molecules. The esterification dehydration reaction pathway and energy barrier are shown in Figure 1, where the reaction energy barrier is 48.1 kJ/mol, RC refers to the reactive complex, TS is the transition state, and PC is the product complex. The three hydroxyl groups of TMP fully reacted with decanoic acid to produce trimethylolpropane decanoate TME-C_10_. It is obvious that the functional groups C=C double bond and β-H are not contained in TME-C_10_ compared to the GT molecular structural formula, which is shown in Figure 2.

**Figure 3:**
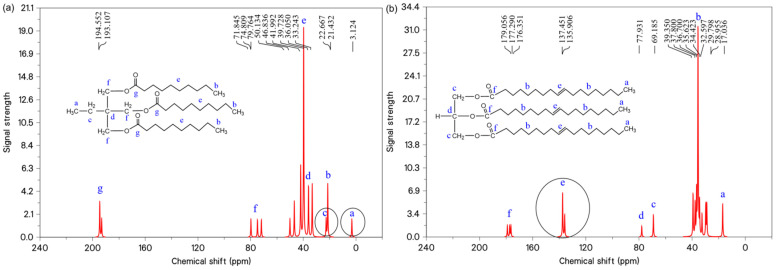
^13^C-NMR spectra: (**a**) TME-C_10_; (**b**) GT.

In order to better express the differences in the major functional groups of each system before and after the modification of the molecular structure, the molecular models were further discriminated based on nuclear magnetic resonance carbon spectroscopy (^13^C-NMR). The ^13^C-NMR spectra of the TME-C_10_ and GT systems are shown in Figure 3, where the spectra indicate that the TME-C_10_ molecule has 14 carbon signals and the GT molecule has 16 carbon signals. The peaks labeled a~g in the figure serve to identify specific functional groups within the molecule, facilitating precise correlation between the functional groups and their respective carbon signals. By analyzing the chemical shift information in ^13^C-NMR, it can be seen that the chemical shifts in the TME-C_10_ molecule have two peaks at 3.124 ppm and 22.667 ppm, which are the ethyl group substituted for β-H. Compared with the GT molecule, no functional group signal of carbon–carbon double bonds was observed, which has been highlighted in the figure.

### 2.2. Molecular Orbitals and Density of States

**Figure 4:**
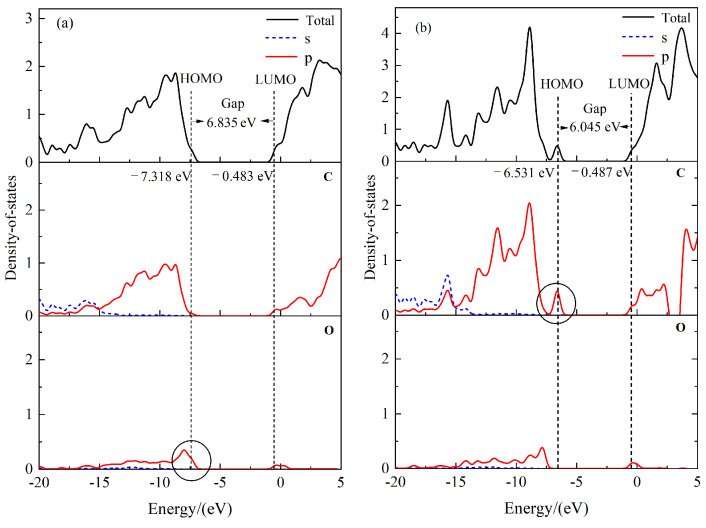
The molecular orbitals and density of states: (**a**) TME-C_10_; (**b**) GT.

**Figure 5:**
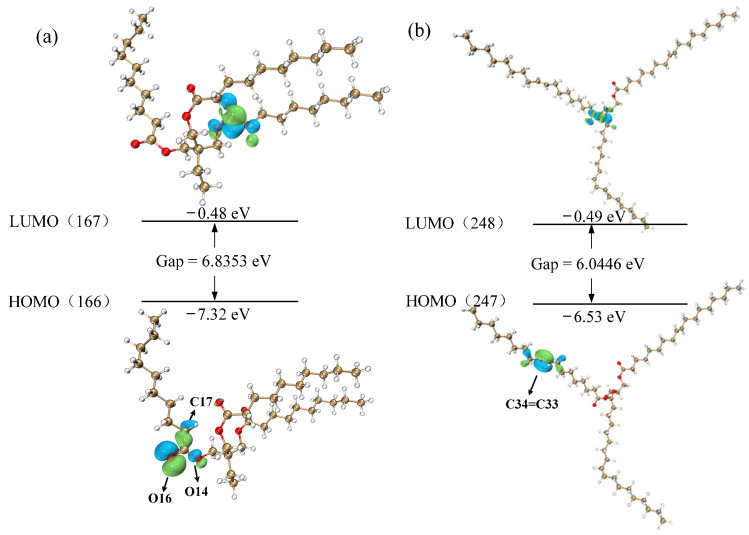
Isosurface maps and energy levels of the molecules HOMO and LUMO: (**a**) TME-C_10_; (**b**) GT.

By combining the frontier orbital energy level distribution and to better understand the local energy states of the molecular systems, we utilized the Multiwfn [24] to calculate and plot the density of states (DOS) for the TME-C_10_ and GT molecular structures, as shown in Figure 4. The figure presents the total DOS (TDOS) and partial DOS (PDOS) for C and O atoms together, with dashed lines highlighting the positions of the HOMO and LUMO. From the trend in the change in each curve, it can be judged that basically all of them are contributed by C-atom MOs, especially in the orbitals in the lower energy interval, such as the PDOS of C atoms of the two systems near −18 eV, which is very close to the TDOS. Additionally, it can be seen that in the PDOS curves, the TDOS of TME-C_10_ and GT are mainly contributed by the 2P MOs of C and O atoms. Comparison of the density-of-state diagrams for TME-C_10_ and GT reveals that the largest difference between the two is in the HOMO orbitals, as observed in Figure 4a. The HOMO orbitals of the TME-C_10_ molecule are mainly constituted by the 2p orbitals of the O atoms, with a high contribution of 92.45%, of which the O16 atoms dominate with a contribution of 65.98%, which is also evident from combining with the HOMO orbitals given in Figure 5a, where the isosurfaces can also be seen. The HOMO shown in Figure 4b is mainly composed of the 2p MOs of C atoms with a contribution of 89.25%, in which the sum of C33 and C34 atoms contributes 77.31%, and the HOMO isosurfaces shown in Figure 5b are also concentrated on C33=C34.

The isosurface plots of the highest occupied molecular orbital (HOMO) and the lowest unoccupied molecular orbital (LUMO) as well as the energies of the two molecular systems, TME-C_10_ and GT, are given in Figure 5. From the figure, it can be seen that the HOMO energies are −7.32 eV and −6.53 eV, respectively, which are both negative, indicating that the TME-C_10_ and GT structures are stable under normal conditions. Observation of the orbital isosurfaces shows that both HOMO and LUMO of TME-C_10_ are distributed on the ester group (-COOC); whereas, the HOMO of GT is distributed on the C=C bond and its LUMO is distributed on -COOC. After molecular orbital and density-of-states analysis, the differences between the molecular structures of TME-C_10_ and GT are mainly reflected in the HOMO distributions of the two systems, and it is obvious from Figure 4 and Figure 5 that the different HOMO energy values are the main reasons for the different sizes of the HOMO-LUMO gap energy values of the two molecular systems, and the computed HOMO-LUMO gap values are 6.835 eV and 6.045 eV, respectively.

### 2.3. Influence of External Electric Fields on the Dipole Moment and Polarizability of Molecular Systems

**Figure 6:**
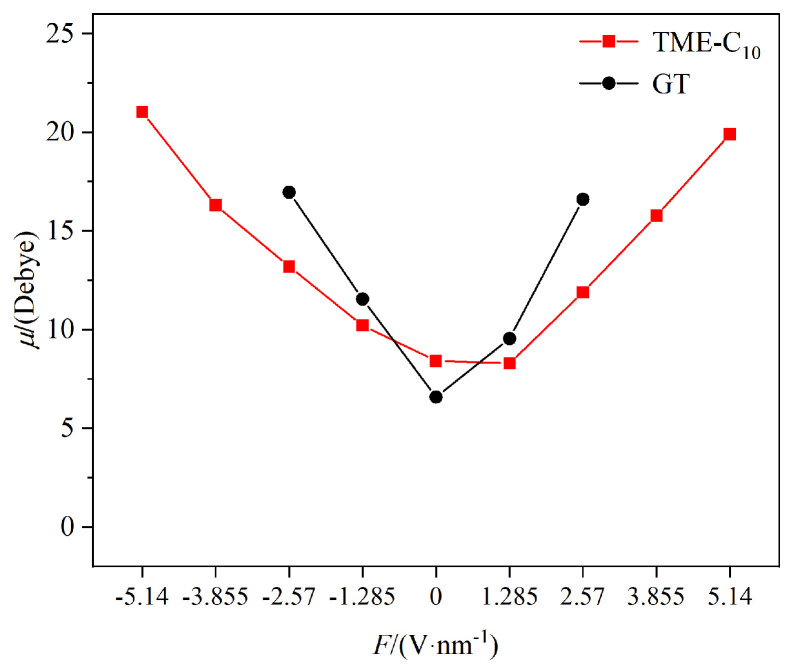
Variation in the molecular dipole moment with electric field strength.

The dipole moment can be used to characterize the polarity of molecules. The dipole moments of the TME-C_10_ and GT molecules under an external electric field were calculated, and the results are shown in Figure 6. As the electric field strength increases, the dipole moment of the two molecules significantly increases. Except for the intrinsic dipole moment, at the same electric field strength, the dipole moment of the GT molecules is greater than that of the TME-C_10_ molecules, indicating that the GT molecules are more easily polarized. The molecular dipole moment can characterize the spatial configuration of the molecules. Since the GT molecular system has higher polarity and is more sensitive to the electric field under the same electric field strength, the molecular system becomes unstable and dissociates when the electric field strength exceeds 2.57 V/nm. Consequently, data points are missing for both the dipole moment and ionization energy calculations of the GT molecule under the influence of electric fields in both positive and negative directions, and the results are only displayed within the range of −2.57 V/nm to 2.57 V/nm. Compared to the GT molecule, under the same electric field strength, the dipole moment of the TME-C_10_ molecule is generally smaller in magnitude than that of the GT molecule, indicating that the TME-C_10_ molecule has better stability under the applied field strength.

Polarizability and hyperpolarizability are important parameters reflecting the response of molecular systems to an external electric field; the static polarizability *α* and the first hyperpolarizability *β* of the TME-C_10_ and GT systems were calculated by using the DFT, and the results of the calculations are shown in Table 1.

As can be seen from Table 1, the GT molecule exhibits a large polarizability and significant polarization anisotropy, with the *α*_xx_ and *α*_yy_ components being similar in magnitude and significantly larger than the *α*_zz_ component. When a static electric field of 2.57 V/nm is applied along the Z-axis, the dipole moment of the GT molecule is 8.028 D, which is smaller than the dipole moment of 15.1430 D when the same electric field is applied along the X-axis, further confirming the polarization anisotropy characteristics of the GT molecule. To determine the insulation performance of a dielectric, the most unfavorable factors often need to be considered. Therefore, the dielectric strength of the TME-C_10_ and GT systems is still analyzed based on the calculation results when an electric field is applied along the X-axis direction.

**Figure 7:**
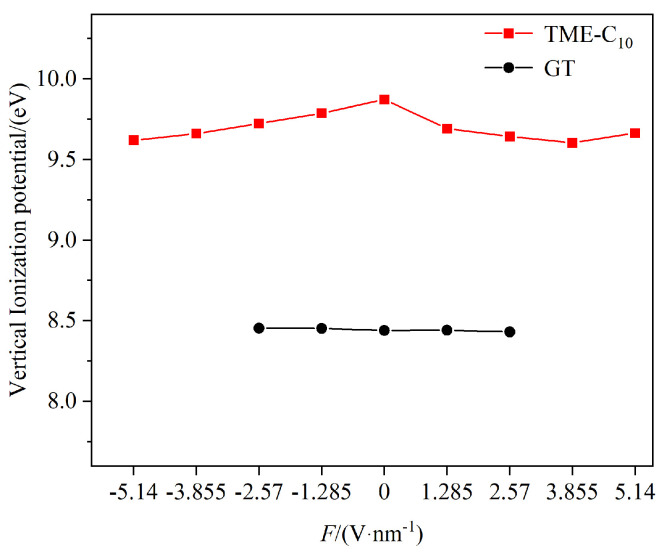
Variation in molecular ionization energy with electric field.

Scholar K.P. Brand demonstrated that the dielectric strength of a molecular system is related to two basic characteristics of the molecule, namely, the polarizability and the ionization energy [25]. Both the TME-C_10_ and GT molecules have a positive β_tot_, which indicates that their overall polarizability increases under the action of an external electric field, with the polarizability of the medium-chain fatty acid triglyceride, TME-C_10_, being more affected by the electric field. Figure 7 shows the relationship between the ionization energies of the TME-C_10_ and GT molecules with the applied electric field; overall, the ionization energies of the two molecules fluctuate less with the increase in the electric field strength, and the ionization energy of the TME-C_10_ molecule is larger than that of the GT molecule. According to the relationship [26] between the reference gas dielectric strength *E*r, polarizability *α*, and ionization energy *ε*, expressed as *E*r = 0.001159*α*^1.175^*ε*^1.776^, it is evident that the dielectric strength increases with the enhancement of the medium’s ionization energy and average polarizability. Clearly, the newly synthesized ester TME-C_10_ molecule exhibits stronger dielectric strength and superior insulation performance.

### 2.4. Micro-Mechanism of Functional Group Regulation of Dielectric Properties

#### 2.4.1. Electron Ionization and Electron Affinity of Molecules

In this section, the ionization energies of the TME-C_10_ and GT molecular systems were calculated separately, and in order to obtain more accurate results, the calculations were finished at the M06-2X/def2-TZVP level, and the results are shown in Table 2. The larger the IP, the larger the energy required for the system to free itself, and the more difficult it is for electrons to escape their bonds, and comparing with the values in the table, it is clear that the IP of TME-C_10_ is slightly larger. Table 2 also provides the EA of TME-C_10_ and GT. In order to calculate the EA energy of the system more accurately, which can better represent the state of the anion in the system, the calculation level adopts a higher level of ωB97XD/aug-cc-pVDZ, and it can be seen from the table that the EA values are all negative, indicating that both systems are unable to capture additional electrons. The fundamental gap (E_fund_, E_IP_ − E_EA_) was also calculated for both systems, where the E_fund_ value for the GT system is smaller, indicating that the electrons in the GT system are more easily polarized.

**Figure 8:**
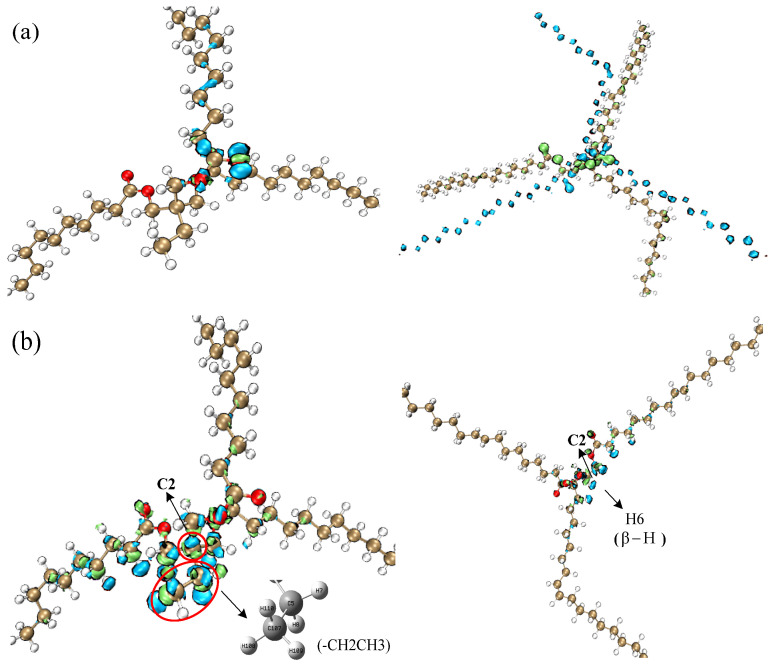
Isosurface maps of electron density at optimized neutral geometry of TME-C10 and GT systems: (**a**) $\Delta \rho =\rho_{N-1}-\rho_{N}$; (**b**) $\Delta \rho =\rho_{N+1}-\rho_{N}$.

The electron distribution in the TME-C_10_ and GT systems during vertical electron ionization and affinity processes can be analyzed using density difference maps. The electron density isosurfaces of the TME-C_10_ and GT systems are given in Figure 8, where $\rho_{N}$, $\rho_{N-1}$, $\rho_{N+1}$ indicate the electron number state of the molecular system in the neutral state, *N* − 1 is the electron density of the system ionized off one electron state, and *N* + 1 is the electron density of the system combined with one electron state, respectively, with blue representing the distribution of holes and green representing the distribution of electrons. Figure 8a shows the electron ionization isosurfaces for the two systems. Observing the blue region, it can be found that the ionized electrons of the TME-C_10_ system mainly come from the O atoms in the ester group, while the ionized electrons of the GT system mainly come from the C atoms in the molecular system. After further increasing the values of the isosurfaces, it is found that the ionization of electrons from the neutral state to the cationic state in the TME-C_10_ and GT systems can be approximated as the loss of electrons from the HOMO. Figure 8b shows the electron affinity reaction isosurfaces for the two systems, and the green area clearly indicates the location of the additional extra electron distribution added during the electron affinity process, which is mainly distributed on the ethyl (-CH2CH3) for the TME-C_10_ system, while the GT system is mainly distributed on the central carbon atom C2 and on the O atom of the ester group. The electron affinity energies given in Table 2 are both negative, already indicating that no further electrons can be obtained for both systems, and even if they are obtained, they are rapidly shed. 

#### 2.4.2. Molecular Vibrations and IR Spectra

**Figure 9:**
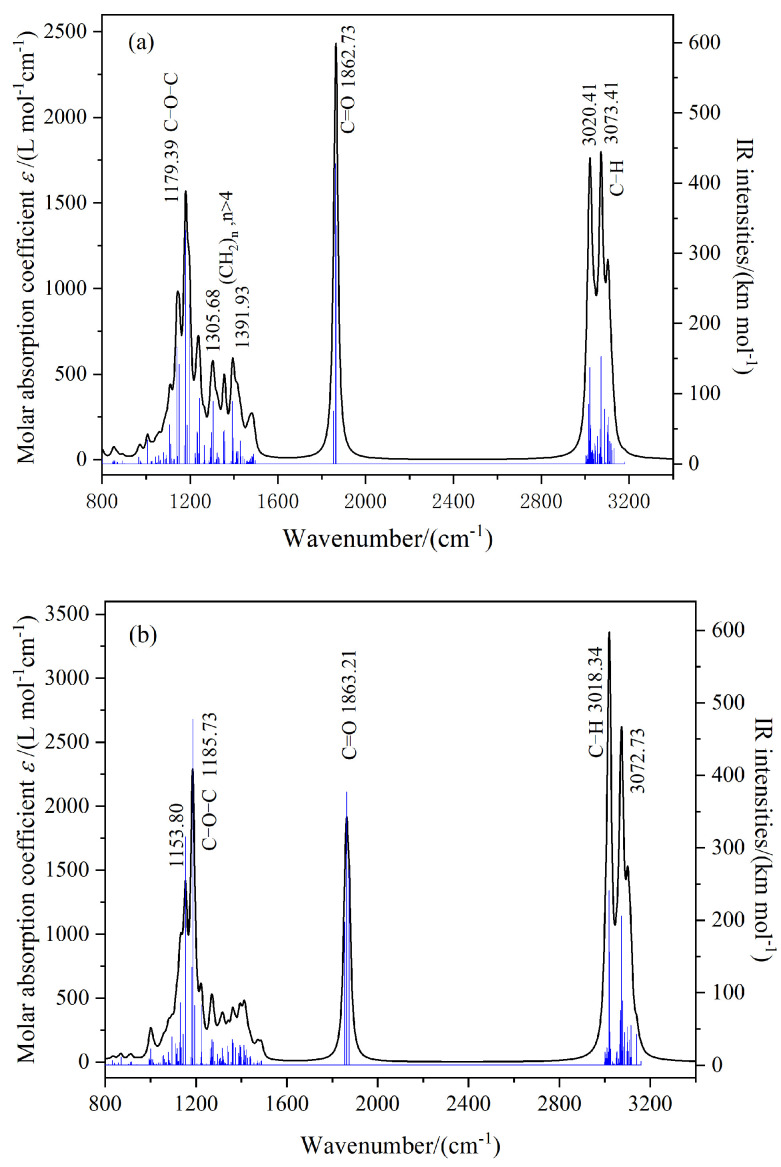
Molecular vibrations and infrared (IR) spectra: (**a**) TME-C_10_; (**b**) GT.

To analyze more comprehensively the molecular structure and properties of TME-C_10_ and GT, molecular vibrational frequency calculations were performed on the basis of the above studies, at the same level as the molecular structure optimization calculations. Figure 9 shows the IR spectra plotted according to the vibrational frequency calculation results, where the black curve corresponds to the molar absorption coefficient and the blue vertical line corresponds to the theoretically calculated infrared intensity. From the figure, several obvious peaks can be seen, including the C-H stretching vibration, C=O stretching vibration, (CH2)n, n > 4 C-H bending vibration, and C-O stretching vibration. The vibration frequencies are marked in the figure. Comparison of Figure 9a,b shows that when the molecular system is irradiated with infrared light, the infrared absorption peaks of the two systems are in the same position, but the infrared intensities are different. However, the infrared intensity of the C=C carbon double bond functional group in the GT system is 0, which is prohibited from transition. Due to the fact that the amplitude of the change in dipole moment of molecules during vibration is proportional to the infrared intensity, it can be judged that the dipole moment of the TME-C_10_ molecule in the ground state is larger than that in the GT molecular system. After calculation, the intrinsic dipole moments $\mu_{0}$ of the two systems were found to be 8.506 D and 6.768 D, respectively. The intrinsic dipole moment of the TME-C_10_ molecule is larger, and the analysis results are consistent with the calculated results shown in Figure 6.

#### 2.4.3. Electronic Excited States of Molecules

**Figure 10:**
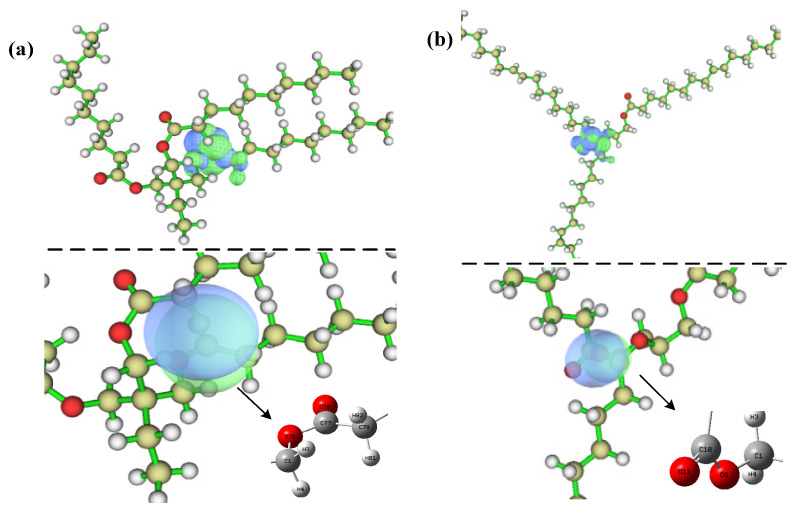
Isosurfaces of hole and electron distribution under S0→S1 excitation: (**a**) TME-C_10_; (**b**) GT.

Electronic excitations are described by orbital transitions, which are transitions of multiple MO pairs with corresponding weighting coefficients. Table 3 shows the parameters of the first excited state S0→S1 of TME-C_10_ and GT, in which $E_{\mathrm{ex}}$ is the excitation energy of the S0→S1 transition, and the numerical magnitude reflects the difficulty of the electron transition; *D* index is a measure of the distance between the center of mass of the hole–electron; *Sr* index is the geometric mean of the overlap of holes and electrons, with larger values indicating a higher degree of overlap and smaller values indicating a more pronounced separation of holes and electrons; *t* index is a measure of the degree of hole–electron separation, and when t<0, it indicates that there is no significant separation of holes and electrons; and Δσ index reflects the difference in the overall spatial breadth of distribution of holes and electrons. Overall, among the parameters of the first excited state, the *D* index is smaller, the *Sr* index is larger, the *t* index is negative, and the Δσ index is also smaller, indicating that there is no obvious change in the distribution area of the electrons before and after excitation in the molecular structure of TME-C_10_ and GT, which is obviously local excitation (LE). The distribution of holes and electrons in the excited states of S0→S1 is shown in Figure 10.

As seen in Figure 10, in the S0→S1 excited state, both holes and electrons of the TME-C_10_ and GT molecules appear only in the ester group part, where blue represents the distribution of holes and green represents the distribution of electrons. The top half of Figure 10a,b shows the hole and electron distribution of the system, and the bottom half shows the smoothed hole–electron distribution describing the isosurfaces [27], where the regions of the hole and electron distribution overlap, and it can be judged that the excitation mode of S0→S1 is LE, which is in agreement with the results deduced from the *D*, *Sr*, *t*, Δσ indices in Table 3. From the comparison of the hole–electron distribution and the excitation-related parameters, it is found that there is little difference between TME-C_10_ and GT in S0→S1 excitation. From the isosurfaces of the hole–electron distribution as well as the excitation-related parameters, it can be seen that the lowest electronically excited states of the TME-C_10_ and GT molecules are not dominated by HOMO→LUMO, and the reaction sites are all on the ester group and are favorable for the compounding of holes and electrons to emit photons, so that the electron-transition intensity needs to be further calculated.

#### 2.4.4. UV-Vis Spectrum

The absence of electric and temperature fields can make the insulating oil molecules freely form mainly light radiation energy caused by photoionization. When the light is incident to the molecular system, the base state system absorbs the corresponding frequency of electromagnetic waves and transitions to the various excited states; when the energy of the photon exceeds the free energy of the system, the molecular system may be free.

**Figure 11:**
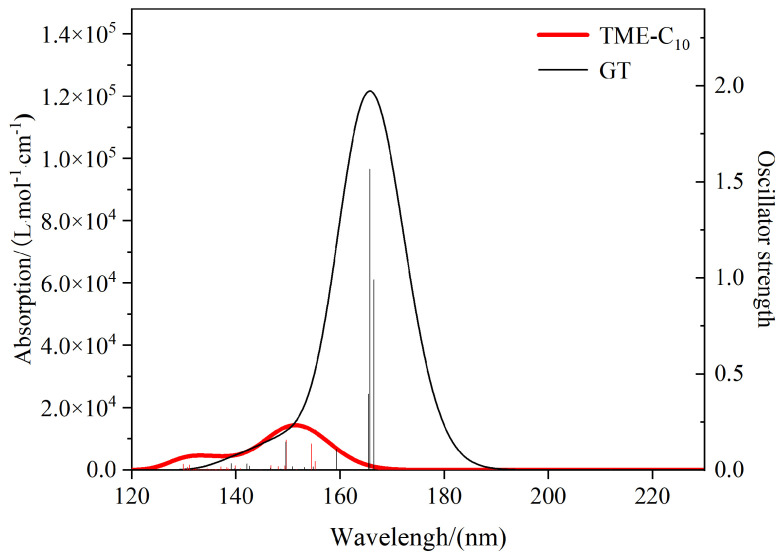
Simulated UV-Vis spectra of the TME-C_10_ and GT systems.

Figure 11 shows the ultraviolet–visible (UV-Vis) spectra plotted based on the excitation energies and vibronic intensities obtained by calculating 50 excited states for the TME-C_10_ and GT molecules. The horizontal coordinate position of the vertical line indicates the magnitude of the excitation energy, the height of the vertical line is the value of the vibronic intensity corresponding to the right-hand axis, and the curve is the molar absorption coefficient of the spreading that corresponds to the left-hand axis. As can be seen from the figure, there is no obvious absorption peak in the visible region (380–760 nm) for TME-C_10_ and GT, indicating that pure TME-C_10_ and GT are colorless. For the TME-C_10_ system, there are two obvious absorption peaks in the UV-Vis curve, and the stronger absorption peak is at 151.47 nm, which comes from the bis-simplex S0→S7 and S0→S5 excitations, with a vibronic intensity of 0.155; whereas, although there is only one absorption peak in the UV-Vis spectra of the GT system, the vibronic intensity of the system is as high as 1.57, with peaks at 165.73 nm and 165.73 nm from the bis-simplified S0→S4 and S0→S5 excitations, respectively. From the plots, it can be seen that the absorption peaks of TME-C_10_ and GT are in the far-ultraviolet region (100~200 nm), and the incidence of this incident wave is very low, which indicates that the two architectures are stable in the absence of interference from the external environment. The oscillator intensity can reflect the strength of the transition between the two states. The larger oscillator intensity indicates that the base state of the system is more likely to absorb the corresponding frequency of electromagnetic waves and excited to an excited state. It is clear that once the conditions permit, GT is more prone to electronic transitions. The wavelengths of the excitation in the S0→S1 of TME-C_10_ and GT are 216.53 nm and 216.71 nm, respectively, due to the molar absorption coefficient being very small, close to 0, and the transition intensity is very low. The extremely low intensity of the transition, close to 0, is not shown in the graph, so the absorption peaks of the first excited state are not seen.

**Figure 12:**
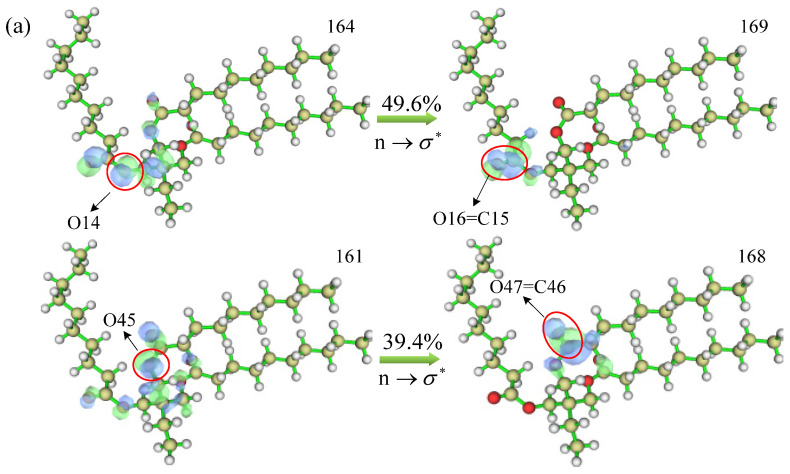

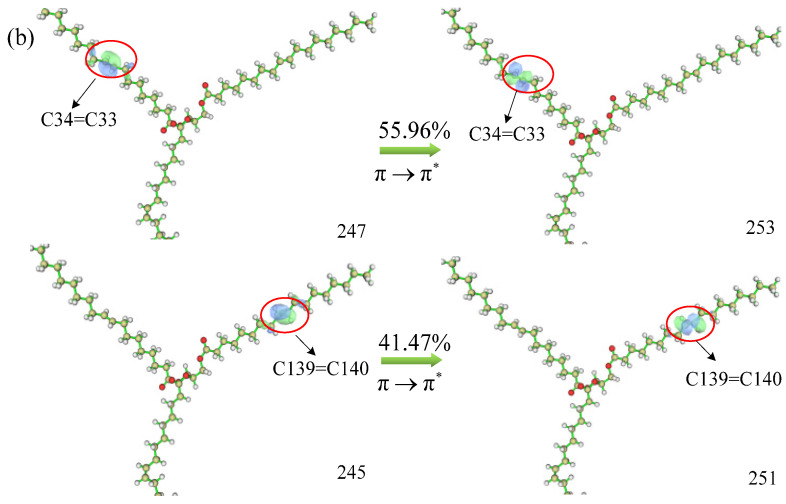
Contribution values of TME-C_10_ and GT to the MO transitions in absorption peaks: (**a**) TME-C_10_; (**b**) GT.

The excitation energies and vibronic intensities of the absorption peaks of the UV-Vis spectra of TME-C_10_ and GT have more obvious differences, especially the vibronic intensities that are nearly 10 times different, and to best explain this phenomenon, the contributions in the MO transitions of the absorption peaks were further calculated, as shown in Figure 12, where blue represents the distribution of holes and green represents the distribution of electrons. From the figure, it can be seen that there are two groups of MO pairs with higher absorption peak transition contributions for the TME-C_10_ molecule, which are MO164→MO169 and MO161→MO168; and there are also two groups of MO pairs with higher absorption peak transition contributions for the GT molecule, which are MO247→MO253 and MO245→MO251. After careful observation, it was found that the upper and lower MO pairs of each system had the same mode of electron transitions. In the TME-C_10_ system, the electron transition occurs on the ester group structure, and increasing the orbital equivalent face value, it is found that the excitation process is the n orbital excitation of the O atom on the C-O bond of the ester group to the $\sigma^{*}$ orbital of the C=O, with the electron transition mode of $n\to \sigma^{*}$; whereas, in the GT system, the electron transition occurs on the C=C bond, which belongs to the typical $\pi \to \pi^{*}$ transition mode. Therefore, the bonding structures of C15=O16 and C46=O47 in Figure 12a and C33=C34 and C139=C140 in Figure 12b are broken. The excitation energy of the absorption peak of the GT system is smaller than that of the TME-C_10_ system, so the wavelength of the absorption peak in the TME-C_10_ system is shorter than that in the TME-C_10_ system, which is also reflected from the electron transition mode.

## 3. Discussion

Natural ester insulating oils are typical ester compounds, and new synthetic esters based on vegetable oils also contain ester functional groups, which do not contain C=C the double bond and β-H functional groups, compared with traditional natural ester insulating oil molecules. In this paper, we explored the microscopic mechanism for the difference in the performance of the TME-C_10_ molecule compared with the GT molecule, and establishes the influence of functional groups (such as C=C double bonds, ester groups, and β-H) on the system’s performance.

Conventional natural ester insulating oils are susceptible to aging due to the presence of unstable C=C double bonds and β-H in the molecules, which affects the service life of transformers. From the results of DFT calculations, the energy gap value of the molecular system changes from 6.045 eV to 6.835 eV, which increases the resistance to electron movement and makes the transition of electrons in the TME-C_10_ molecule to the higher energy levels require greater energy. In addition, under the action of the external electric field, the ionization energy of the TME-C_10_ molecule decreases gradually with increasing electric field strength, but the ionization energy of the TME-C_10_ molecule is larger than the GT molecule under the same conditions, and the value of the $\beta_{\mathrm{tot}}$ parameter of the TME-C_10_ molecule is larger and positive. The polarization rate increases with increasing electric field, and the structure of the GT molecule is difficult to dissociate when the strength of the electric field is larger than 2.57 V/nm. It is difficult to dissociate because the dielectric strength of the insulating material and the system is not as strong as the one for the GT molecule. Since the dielectric strength of insulating materials is closely related to the polarizability and ionization energy of the system, the comprehensive results indicate that the new natural ester TME-C_10_ molecules not only eliminate the influence of unstable functional groups in traditional natural ester molecules, but also exhibit excellent insulating properties.

By viewing the distribution plots of each isosurface of the calculated results for the new natural ester TME-C_10_ molecule and the traditional natural ester GT molecule, it is found that the electronic behavior of the GT molecule is mainly regulated by the C=C double bond, -COOC, and β-H groups, while the TME-C_10_ molecule is related to the -COOC group only, which is a structural difference that leads to the significant difference in the microscopic properties of the two. The HOMO and LUMO of TME-C_10_ are localized on the -COOC group, whereas the HOMO of GT is localized on the C=C bond, and the LUMO is localized on the -COOC group. When the molecular system gains energy, the electronic excitation process in the TME-C_10_ system is characterized by the transition of the n orbital of the O atom in the C-O bond of the ester group to the $\sigma^{*}$ orbital of C=O. In contrast, in the GT system, the electronic transition occurs on the C=C bond, which is a typical $\pi \to \pi^{*}$ transition. The electron affinity values of the TME-C_10_ and GT molecules are −0.37 eV and −0.27 eV, respectively. Further analysis of the electron affinity reaction isosurfaces indicates that the presence of the β-H group in GT molecules makes it easier for them to accept electrons, thus triggering free radical chain reactions at lower energy levels and accelerating the aging of the insulating oil.

## 4. Materials and Methods

The development of quantum chemical theory provides a new method for exploring new high voltage insulating dielectrics. In this paper, we analyze the microscopic properties of molecular structures based on density functional theory (DFT). Unless otherwise stated, all structure optimization calculations involved in this paper are performed using the B3LYP/def2-SVP basis set, which requires dispersion correction by the DFT-D3 method. For single-point energy calculations, the higher-level M06-2X/def2-TZVP is chosen, and it is noted that the basis set must be with the dispersion function when calculating dipole moments, and the generalized functions and basis sets used for all kinds of calculations in the paper mainly refer to the relevant test literature as well as the computational experience [28,29,30]. On this basis, the wavefunction analysis software Multifunctional Wavefunction Analyzer (Mutiwfn) [31] was combined with the visualization software Visual Molecular Dynamics (VMD) [32] to calculate and visualize the molecular front orbital distribution. In addition, using the field keyword, an electrostatic field with a step size of 1.285 V/nm and a range of −5.14 to 5.14 V/nm was added along the positive and negative half-axis of the x-axis to optimize the new synthetic ester molecules with the traditional natural ester molecule system and to calculate the electronic properties for comparing and analyzing the dielectric strength.

In quantum chemical calculations, the electronic structure of a molecular system can be analyzed using molecular orbitals (MO) versus density-of-states (DOS) by applying the relationship shown in Equation (1):

(1) $$\mathrm{TDOS}(E)=\sum_{i}\delta (E-\varepsilon_{i})$$

where E is the energy of the $\varepsilon_{i}$ orbital *i*, and δ refers to the Dirac function. By analyzing the contributions of each atom in the system to the highest occupied molecular orbital (HOMO) and lowest unoccupied molecular orbital (LUMO), we can clarify the nature of the difference in the electronic structural properties between the new synthesized ester molecule and traditional natural ester molecules.

Electron affinity (EA) reflects the energy change in a molecule after obtaining electrons *E*_EA_ = *E*(*N*) − *E*(*N +* 1), with *E*(*N*) and *E*(*N* + 1) representing the energy of neutral molecules and anions, respectively.

When natural ester insulating oil is applied in transformers, it is subjected to an external electric field. By Taylor expansion of the molecular system energy E on the uniform external electric field F, Equation (3) can be obtained.

(2) $$\begin{array}{cc} E(F) & =E(0)+{\left.\frac{\partial E}{\partial F}\right|}_{F=0}F+{\left.\frac{1}{2}\frac{\partial^{2}E}{\partial F^{2}}\right|}_{F=0}F^{2}+{\left.\frac{1}{6}\frac{\partial^{3}E}{\partial F^{3}}\right|}_{F=0}F^{3}+\cdots \\ & =E(0)-\mu_{0}F-\frac{1}{2}\alpha F^{2}-\frac{1}{6}\beta F^{3}-\cdots \end{array}$$

where $\mu_{0}$ is the intrinsic dipole moment of the molecular system, which is the dipole moment in the absence of an external electric field; the polarization rate *α* reflects the change in the dipole moment of the system before and after the application of the electric field, and is the second-order derivative of the system energy *E* with respect to the external electric field *F*; and the first hyperpolarizability rate *β* embodies the non-linear polarization effect, and is the third-order derivative of the system energy *E* with respect to the external electric field *F*.

After the dielectric absorbs external energy, the ground state electrons of its molecules or atoms are excited to higher energy-level orbitals, and the electrons are excited from the ground state to the excited state. The first 50 excited states are calculated separately by using the time-containing density flood theory (TDDFT) on the basis of B3LYP/def2-TZVP, and the nature of the electron leaps of the two molecular systems is analyzed using the hole–electron analysis method [29]. The hole–electron analysis method satisfactorily solves the multiorbital electron-leaping excitation process inside the molecule, whose density distribution is defined as:

(3) $$\begin{array}{l} \rho^{\mathrm{hole}}(r)=\rho_{\left(\mathrm{loc}\right)}^{\mathrm{hole}}(r)+\rho_{\left(\mathrm{cross}\right)}^{\mathrm{hole}}(r)\\ ={\sum_{i\to a}\left(w_{i}^{a}\right)}^{2}\phi_{i}(r)\phi_{i}(r)+\sum_{i\to a}\sum_{j\neq i\to a}\left(w_{i}^{a})(w_{j}^{a}\right)\phi_{i}(r)\phi_{j}(r) \end{array}$$

(4) $$\begin{array}{l} \rho^{\mathrm{ele}}(r)=\rho_{\left(\mathrm{loc}\right)}^{\mathrm{ele}}(r)+\rho_{\left(\mathrm{cross}\right)}^{\mathrm{ele}}(r)\\ ={\sum_{i\to a}\left(w_{i}^{a}\right)}^{2}\phi_{a}(r)\phi_{a}(r)+\sum_{i\to a}\sum_{i\to b\neq a}\left(w_{i}^{a})(w_{i}^{b}\right)\phi_{a}(r)\phi_{b}(r) \end{array}$$

where r is the coordinate vector, ϕ is the orbital wave function, i or j is the occupied orbital labeling, a or b is the empty orbital labeling, w is the group coefficient of the group function, and both the hole distribution $\rho^{\mathrm{hole}}$ and the electron distribution $\rho^{\mathrm{ele}}$ are divided into two parts: the local term *loc* and the cross term *cross.*

When the applied electric field strength is large, it leads to the molecules of one or several electrons removing the binding of the nucleus and the formation of free electrons and positive ions, that is, the insulating oil molecules undergo ionization. The energy that can cause the ground-state electrons to detach from the system is called the ionization potential IP. *E*_IP_ = *E*(*N* − 1) − *E*(*N*), where *E*(*N*) and *E*(*N* − 1) are the energies of neutral molecules and cations, respectively.

In this article, we evaluate the feasibility of the esterification reaction between TMP and C_10_H_20_O_2_ by calculating the intrinsic reaction coordinate (IRC). The IRC search identifies the transition states (TS) and intermediate states (IM) between reactants and products, and constructs the energy change pathway of the esterification reaction, where the product is the initial model of the novel natural ester molecular structure. In the search for TS in esterification reactions, the B3LYP method was used in combination with the 6–31 G (d, p) basis set for structural optimization and frequency calculation. When using the keyword ‘IRC’ to calculate the reaction path, it is important to note that the calculation level must be strictly consistent with the TS search, and to use the keyword ‘calcfc’ to generate a more accurate Hessian matrix. Based on the initial model of IRC prediction, we compared and analyzed the new natural ester molecule with the traditional glycerol trioleac (GT) acid molecule and obtained parameters for the two systems at the same computational level, such as the molecular orbitals, total energy, dipole moment, ionization energy, polarizability, and electron affinity. Further, we evaluated the microscopic properties of the novel natural ester molecules at the molecular level, delving into the mechanism by which the functional groups regulate the molecular properties.

## 5. Conclusions

This study employs molecular simulation technology to analyze the influence mechanisms of unstable groups in traditional natural ester molecules on system performance. Based on this analysis, a novel natural ester molecular structure is proposed. Through molecular structure modification, the negative impact of unstable groups has been effectively reduced, significantly enhancing the overall performance of insulating oil.

(1) Compared to the traditional GT molecule, the new synthetic ester insulating oil TME-C_10_ molecule does not contain C=C double bonds or β-H groups, effectively eliminating the influence of unstable groups in traditional natural ester molecules and significantly improving the chemical stability and anti-aging performance of the insulating oil.

(2) The differences in molecular structure directly affect their electronic behavior and energy level characteristics. The TME-C_10_ molecule has a larger HOMO-LUMO gap and unique electron distribution characteristics, resulting in higher energy requirements for electron transitions, demonstrating stronger electron binding capacity and higher molecular stability.

(3) Under the influence of an external electric field, the ionization energy of the TME-C10 molecule decreases with increasing electric field strength, but its ionization energy remains higher than that of the GT molecule, indicating that the TME-C_10_ molecule has higher dielectric strength and resistance to electric field breakdown. Comprehensive performance analysis shows that the TME-C_10_ molecule is a new insulating oil material with superior performance compared to the traditional GT molecule, demonstrating broad application prospects.

## Figures and Tables

**Table 1:** Static polarizabilities and hyperpolarizabilities of TME-C_10_ and GT.

	$\alpha_{\mathrm{xx}}/$.a.u	$\alpha_{yy}/$.a.u	$\alpha_{\mathrm{zz}}/$.a.u	$\alpha_{\mathrm{iso}}/$.a.u	Δα/.a.u	$\beta_{\mathrm{tot}}/$.a.u
TME-C_10_	527.476	477.802	434.104	479.794	85.727	266.15
GT	845.895	830.492	623.585	766.657	215.984	127.30

**Table 2:** Electron vertical ionization energies and electron vertical affinity energies of TME-C_10_ and GT.

	VIP/eV	VEA/eV	E_fund_/eV
TME-C_10_	9.87	−0.37	10.24
GT	8.44	−0.27	8.71

**Table 3:** Various parameters of TME-C_10_ and GT excited states S0→S1.

	$E_{\mathrm{ex}}/$eV	D/Å	Sr/a.u.	t/Å	Δσ/Å
TME-C_10_	5.726	0.661	0.51705	−0.266	0.165
GT	5.721	0.512	0.51712	−0.97	0.147

## Data Availability

Data is contained within the article.
